# Chronic Stress Modulates Interneuronal Plasticity: Effects on PSA-NCAM and Perineuronal Nets in Cortical and Extracortical Regions

**DOI:** 10.3389/fncel.2019.00197

**Published:** 2019-05-07

**Authors:** Ana Paula Pesarico, Clara Bueno-Fernandez, Ramón Guirado, María Ángeles Gómez-Climent, Yasmina Curto, Hector Carceller, Juan Nacher

**Affiliations:** ^1^Neurobiology Unit, Program in Neurosciences and Interdisciplinary Research Structure for Biotechnology and Biomedicine (BIOTECMED), Universitat de València, Burjassot, Spain; ^2^Spanish National Network for Research in Mental Health, Centro de Investigación Biomédica en Red de Salud Mental (CIBERSAM), Madrid, Spain; ^3^Fundación Investigación Hospital Clínico de Valencia, INCLIVA, Valencia, Spain

**Keywords:** chronic stress, perineuronal net, PSA-NCAM, medial prefrontal cortex, basolateral amygdala, reticular thalamic nucleus, habenula, hippocampus

## Abstract

Chronic stress has an important impact on the adult brain. However, most of the knowledge on its effects is focused on principal neurons and less on inhibitory neurons. Consequently, recent reports have begun to describe stress-induced alterations in the structure, connectivity and neurochemistry of interneurons. Some of these changes appear to be mediated by certain molecules particularly associated to interneurons, such as the polysialylated form of the neural cell adhesion molecule (PSA-NCAM) and components of the perineuronal nets (PNN), specialized regions of the extracellular matrix. These plasticity-related molecules modulate interneuronal structure and connectivity, particularly of parvalbumin expressing basket interneurons, both during development and adult life. These inhibitory neurons are specially affected after chronic stress and in some stress-related disorders, in which the expression of PSA-NCAM and certain components of PNN are also altered. For these reasons we have decided to study PSA-NCAM, PNN and parvalbumin expressing interneurons after 10 days of chronic restraint stress, a time point in which its behavioral consequences are starting to appear. We have focused initially on the medial prefrontal cortex (mPFC), basolateral amygdala (BLA) and hippocampus, regions affected by stress and stress-related psychiatric diseases, but we have also explored the habenula and the thalamic reticular nucleus (TRN) due to the important presence of PNN and their relationship with certain disorders. PSA-NCAM expression was increased by stress in the stratum lacunosum-moleculare of CA1. Increases in parvalbumin immunoreactive cells were detected in the mPFC and the BLA, but were not accompanied by increases in the number of parvalbumin expressing perisomatic puncta on the somata of principal neurons. The number of PNN was also increased in the mPFC and the habenula, although habenular PNN were not associated to parvalbumin cells. Increased expression of parvalbumin and components of PNN were also detected in the TRN after chronic restraint stress, revealing for the first time substantial effects on this region. Our study shows that, even a short chronic stress protocol, can induce consistent changes in interneuronal plasticity-related molecules in cortical and extracortical regions, which may represent initial responses of inhibitory circuits to counteract the effects of this aversive experience.

## Introduction

Stress, a prevalent experience in modern society, is a major predisposing and triggering factor to mood disorders in humans. Studies in both humans and rodents show that stress impairs the functioning of the medial prefrontal cortex (mPFC) and basolateral amygdala (BLA) (Shepard et al., 2016; Pesarico et al., 2017; Sachs et al., 2018), which are two of the brain regions predominantly dysfunctional in stress-related psychiatric disorders, such as generalized anxiety, major depression and post-traumatic stress disorder (PTSD) (Drevets et al., 2008). The effects of stress and stress-related disorders are not, however, restricted to these two regions and have been also detected in the hippocampus or the habenula, among others (Jacinto et al., 2017; Belleau et al., 2018). Several studies have demonstrated the existence of profound changes in the structure and connectivity of excitatory neurons in the aforementioned regions after chronic stress (McEwen, 2000; Roozendaal et al., 2009; Radley et al., 2015; Jacinto et al., 2017). More recently, it has become evident that chronic stress also induces changes in inhibitory circuits of the adult brain and that similar alterations can be found in patients suffering from major depression or PTSD. At the cellular level these alterations include decreases in the density (Czeh et al., 2005, 2015) and changes in the structure (Gilabert-Juan et al., 2011, 2013, 2017) of certain interneuronal subpopulations. Interestingly, one group of interneurons specially affected by chronic stress are those expressing parvalbumin (PV): decreases in the density of PV+ interneurons have been detected in the mPFC and the hippocampus of adult rats submitted to chronic stress (Czeh et al., 2005, 2015, 2018; Zadrozna et al., 2011; Banasr et al., 2017). Moreover, a reduction of synaptic release has been described in the perisomatic region of mPFC pyramidal neurons, where most of these PV+ interneurons establish their synaptic contacts (Czeh et al., 2018). However, it is not known whether chronic stress could induce changes in this population of interneurons in other brain regions affected in stress-related disorders, such as the amygdala or the habenula.

The maturation and plasticity of PV+ interneurons are mediated by the presence of molecules that influence their structure and connectivity. Recently, different laboratories, including our own, have been studying the role of the polysialylated form of the neural cell adhesion molecule (PSA-NCAM) and of perineuronal nets (PNN) on the plasticity of PV+ interneurons. PSA-NCAM is expressed by a subpopulation of interneurons in the adult telencephalon, and particularly it is present in some of the boutons that PV+ basket cells establish on the somata of pyramidal neurons (Castillo-Gomez et al., 2011). The expression of PSA-NCAM is critical for the regulation of this inhibitory input, both during their development (Di Cristo et al., 2007) and during adulthood (Castillo-Gomez et al., 2011, 2016). PNN are specialized regions of the extracellular matrix, which are particularly abundant surrounding the somata, dendrites and proximal axon segment of fast-spiking, PV+ interneurons (Hartig et al., 1992). Although the function of PNN has not yet been fully resolved, research conducted has suggested that these structures are involved in the closure and reopening of critical periods, response to aversive experiences such as fear and the regulation of synaptic plasticity (Gogolla et al., 2009; Carulli et al., 2010; Pantazopoulos and Berretta, 2016; Sorg et al., 2016; Banerjee et al., 2017).

These important roles of PSA-NCAM and PNN in the development and plasticity of interneuronal circuits are probably reflected in the alterations of their expression levels found in different psychiatric disorders (Varea et al., 2012; Berretta et al., 2015). Moreover, the study of rodent models of chronic stress has revealed alterations in the expression of PSA-NCAM and some of the molecules integrated in the PNN, which may suggest their involvement on the plasticity associated to this aversive experience, particularly on that of inhibitory neurons (Sandi, 2004; Nacher et al., 2013; Soleman et al., 2013). Unfortunately, the information on the expression of these plasticity-related molecules after stress is still fragmentary. The expression of these molecules has not been studied yet in some regions of the adult CNS after chronic stress and most of the information regarding changes in PNN has been obtained in models subjected to stress in early life (Castillo-Gomez et al., 2017; Ueno et al., 2018).

The main objective of the present study was to study the effects of a short period of chronic stress, 10 days of restraint, on PV+ interneurons, PSA-NCAM and PNN in the brain of adult rats. We have focused on the effects on the mPFC and the BLA, because in these regions previous reports already have shown alterations in interneurons after chronic stress, but we have also explored other regions affected by this aversive experience, such as the hippocampus and the habenula. We have also studied the thalamic reticular nucleus (TRN), a region with a very high density of PNN surrounding PV+ interneurons. Interestingly, the density of both PV+ interneurons and PNN is altered in this thalamic region in patients suffering from schizophrenia and bipolar disorder (Steullet et al., 2017), two psychiatric disorders in which stress may act as a precipitating factor.

## Materials and Methods

### Animals and Stress Procedure

Adult male Sprague-Dawley rats (13 weeks-old at the beginning of the experiment; 381.9 ± 4.1 g; Harlan Interfauna Iberica S.L., Barcelona, Spain) were used for all experimental procedures. Animals were housed in groups of 3 in a temperature- and humidity-controlled environment and maintained on a 12 h light/dark cycle with food and water available *ad libitum*. Rats were allowed to habituate to our facilities 1 week prior to the start of the experiments. All experiments were performed during the lights on period, and were conducted in accordance with the Directive 2010/63/EU of the European Parliament and of the Council of 22 September 2010 on the protection of animals used for scientific purposes and was approved by the Committee on Bioethics of the Universitat de València. Every effort was made to minimize the number of animals used and their suffering.

Twelve rats were used for repeated restraint stress experiment. One group of rats (*n* = 6) were stressed 6 h per day (10:00 a.m.) for 10 consecutive days. These rats were kept inside wire mesh restrainers that were clamped at both openings and were placed inside their home cages during the restraint sessions. Six rats were left undisturbed in their home cages and served as controls for this experiment. The control and stressed rats were kept in separate cages. The animals were euthanized 24 h after the last stress session in a random order, in a different room than the one in which restraints were carried out.

### Perfusion, Microtomy, and Immunohistochemistry

Animals were deeply anesthetized with sodium pentobarbital and transcardially perfused with 4% paraformaldehyde solution in phosphate buffer (PB) 0.1 M. The brains were cryoprotected (30% sucrose in PB 0.1 M, 48 h) and afterward cut in 50-μm-thick coronal sections, using a freezing-sliding microtome (LEICA SM2000R, Leica). Sections were collected in 10 subseries.

Two subseries of sections were processed “free-floating” for immunohistochemistry. Briefly, sections were washed in PBS and then incubated for 1 h in 10% normal donkey serum (NDS; Abcys) in PBS with 0.2% Triton X-100 (PBST; Sigma-Aldrich). Afterward, they were incubated for 48 h at 4°C with the appropriate primary antibody or antibody cocktail (see Table 1) diluted in PBST and 5% NDS: (a) a double staining with an antibody against parvalbumin (PV) and a biotin-conjugated lectin from *Wisteria floribunda agglutinin (*WFA) to label PNN and (b) a triple immunostaining using an anti-CaMKII-α primary antibody in combination with anti-PV and anti-synaptophysin (SYN) primary antibodies, to study the perisomatic innervation of pyramidal neurons. After washing, sections were incubated for 2 h at room temperature with appropriate fluorochrome conjugated secondary antibodies or avidin (see Table 1), which were also diluted in PBST. Finally, sections were washed in PB 0.1 M, mounted on slides and coverslipped using fluorescence mounting medium (Dako).

**Table 1:** List of primary and secondary antibodies used in the study.

	Dilution	Company
**Primary antibodies**		
Polyclonal guinea pig anti-parvalbumin	1:2000	Synaptic Systems
WFA lectin biotin-conjugated	1:200	Sigma
Polyclonal rabbit anti- synaptophysin	1:500	Millipore
Monocolonal mouse anti-CaMKII	1:500	Abcam
**Secondary antibodies**		
Goat anti-mouse A635-conjugated	1:200	Life Technology
Goat anti-guinea pig A555-conjugated	1:200	Life Technology
Donkey anti-rabbit A488-conjugated	1:200	Invitrogen
Avidin A647-conjugated	1:200	Invitrogen

Another subseries of sections was processed “free-floating” for conventional immunohistochemistry as follows. Briefly, sections were incubated for 1 min in an antigen unmasking solution (0.01 M citrate buffer, pH 6) at 100°C. After cooling down the sections to room temperature, they were incubated with 10% methanol, 3% H_2_O_2_ in phosphate buffered saline (PBS) for 10 min to block endogenous peroxidase activity. After this, sections were treated for 1 h with 5% NDS (Jackson Laboratories) in PBS with 0.2% Triton-X100 (Sigma) and were incubated overnight at room temperature in mouse monoclonal Men-B anti-PSA-NCAM antibody (1:1400; Abcys). After washing, sections were incubated for 30 min with donkey anti-mouse IgM o donkey anti-mouse IgG biotinylated antibodies (Jackson Laboratories, 1:250), followed by an avidin–biotin-peroxidase complex (ABC, Vector Laboratories) for 30 min in PBS. Color development was achieved by incubating with 3,3′ diaminobenzidine tetrahydrochloride (DAB, Sigma) for 4 min. PBS containing 0.2% Triton-X100 and 3% NDS was used for primary and secondary antibodies dilution. All of the sections studied passed through all procedures simultaneously, to minimize any differences from immunohistochemical staining itself.

### Quantification of PSA-NCAM Expression

We determined PSA-NCAM immunoreactivity intensity in the neuropil of the different regions studied [superficial and deep layers of cingulate 1 (Bregma 3.7 to Bregma –1.4), prelimbic (Bregma 4.7 to Bregma 2.2) and infralimbic (Bregma 3.2 to Bregma 2.2) cortices, BLA (Bregma –1.6 to Bregma –4.16) and all layers in the CA1 region of the ventral (Bregma –4.3 to Bregma –6.3) and dorsal (Bregma –1.6 to Bregma –4.3) hippocampus] using a previously described methodology (Varea et al., 2007). We analyzed 7 slices per animal for the mPFC, 2 for the BLA and 3 for the ventral and dorsal hippocampus. Sections were examined under bright-field illumination, homogeneously lighted and digitalized using a CCD camera. Photographs were taken at 20× magnification. The different regions of interest were delineated using the polygon selection tool in free Java image- processing program Fiji (Schindelin et al., 2012). Gray levels were then converted to optical densities (OD). In order to normalize the values, the gray levels obtained from photographs of the corpus callosum in each section were subtracted from those obtained in the different layers. We used a parallel subseries of section stained with toluidine blue in order to better delineate the regions, subregions and layers under study. We were able to distinguish the border of CA1/CA2 region with the aid of our subseries of sections stained with WFA. This lectin labels intensely the neuropil of the CA2 region making it easily discernible (Lensjo et al., 2017).

### Quantification of PV Expressing Cells and PNN

The total number of PV+ neurons, PNN, and PV+ neurons surrounded by PNN from the hippocampus, BLA, habenula and mPFC (including the cingulate 1, prelimbic and infralimbic cortices) were estimated using a modified version of the fractionator method (West, 1993; Nacher et al., 2002; Varea et al., 2007). We counted cells covering 100% of the sample area, that is, within each section, all labeled cells in the different subdivision and layers were counted. The fractionator sampling scheme refers to the methodology of examining one out of every 10 brain sections. Thus, our modification of the optical dissector combined with a 1:10 fractionator sampling is truly a modification of the optical fractionator method. 1:10 systematic-random series of sections covering the whole rostral to caudal extension of the structures were viewed on an Olympus CX41 microscope. The volume of the different regions analyzed was determined for each animal using the Cavalieri’s principle (Gundersen and Jensen, 1987) and no differences between groups were observed.

Cell somata were identified and counted with a 40× objective using a fluorescence microscope (Olympus CX41). Cells appearing in the upper focal plane were omitted to prevent counting cell caps. In order to classify a cell as positive for PNN or PV every somata was surrounded by a polygon selection using FIJI. Then we analyzed the mean gray value of this selection. Only cells with values higher than 60 were considered positive.

### Quantification of PV and PNN Fluorescence Intensity in the Thalamic Reticular Nucleus and the CA2 Region of the Hippocampus

The high density of PV+ somata and PNN in the TRN made impossible the counting of individual neurons in these structures. Therefore, we measured the intensity of their fluorescence in the reticular thalamic nucleus in order to have an estimation of their abundance and the expression of these markers in this region. We also applied this methodology in the CA2 region, where no individually discernible PNN could be found; WFA labeling was very intense and widespread. Three slices per animal containing the TRN (Bregma –1.3 to Bregma –3.8) and 3 containing the dorsal hippocampus (Bregma –1,7 to Bregma –2.18) were studied. Photographs were taken at 20× magnification with a confocal microscope (Leica TCS SPE). The TRN and the CA2 region were delineated using the polygon selection tool in Fiji (Schindelin et al., 2012). In order to normalize the values, the gray levels obtained from photographs of the external capsule in each section were subtracted from those obtained in the different layers.

### Quantification of Perisomatic Puncta on Pyramidal Neurons

The density of puncta expressing SYN and PV surrounding principal neuron somata (identified by CaMKII-α expression) was analyzed in the regions showing alterations in the number of PV+ cells. For this purpose, we followed a previously described protocol (Guirado et al., 2014). In the mPFC and BLA, between 20 and 25 pyramidal neurons were imaged per animal in three different sections. Confocal z-stacks covering the whole depth of the neuron somata were taken with 0.5 μm step size using a 63× oil objective with 2× digital zoom magnification (Leica TCS SPE confocal microscope). The profile of the soma of these neurons was drawn manually, then a macro was used to analyze the density of puncta around the perimeter. The manual selection was enlarged 1 μm in order to define the perisomatic area. Images were then processed to binarize the 5% brightest elements of the histogram. All particles displaying an area not smaller than 0.15 μm^2^ and not larger than 2.5 μm^2^ were defined as puncta (Di Cristo et al., 2007). The definition of puncta was based on previous literature (Di Cristo et al., 2007). We analyzed areas acquired by the confocal microscope that were averaged 3 times (each confocal plane), decreasing noise significantly. The smallest puncta we recognized had a lateral size of 0.38 μm, which is above the lateral resolution of the confocal microscope.

### Statistics

All slides were coded prior to quantitative analysis, and the code was not broken until the quantification was completed. All experimental results are given as the mean ± S.E.M. First, we evaluated the normality of data using the D’Agostino and Pearson omnibus test. Comparisons between control and stressed groups were performed by the Student’s *t*-test. Probability values less than 0.05 (*p* < 0.05) were considered as statically significant. The correlation coefficients between the number of PV positive neurons and that of PNN in different brain regions, in control and stressed animals), were tested by using Pearson’s correlation analysis. A *p* value <0.05 was considered statistically significant.

## Results

### Effects of Restraint Stress on PSA-NCAM Expression

**FIGURE 1:**
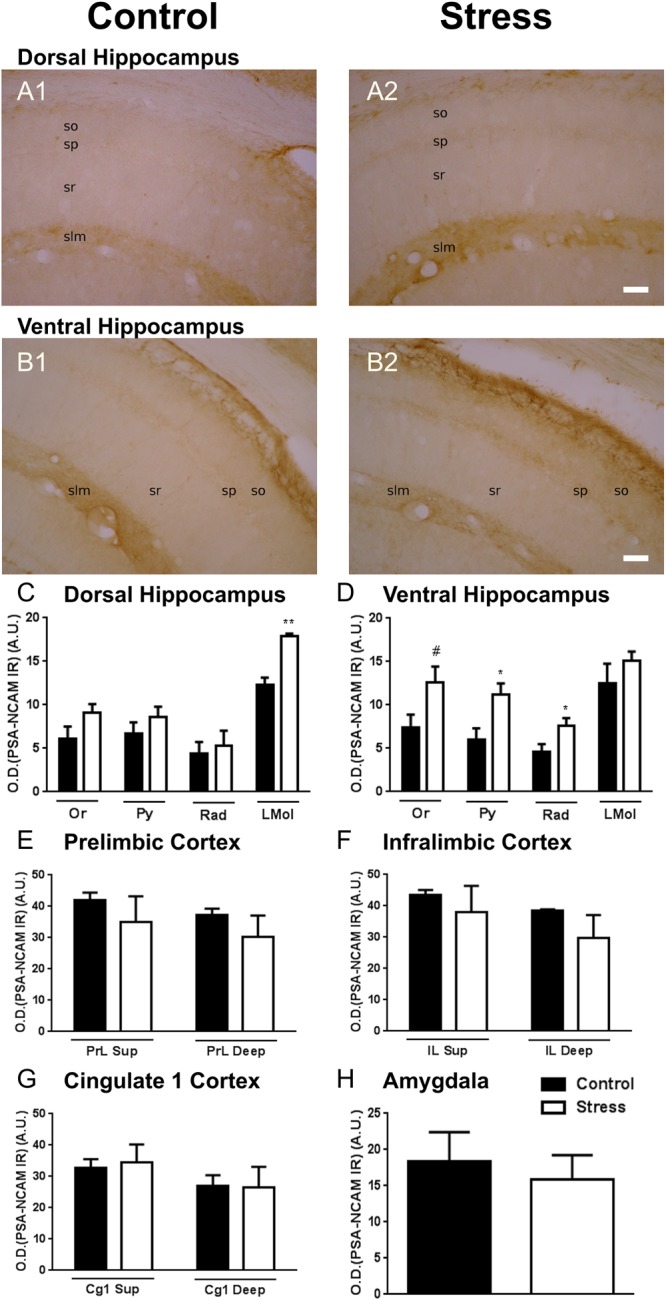
Graphs representing the changes in the intensity of the polysialylated form of the neural cell adhesion molecule (PSA-NCAM) immunostaining in the neuropil of different telencephalic regions. **(A,B)** Microphotographs of the dorsal **(A)** and ventral **(B)** hippocampus of a control **(A1,B1)** and a chronically stressed **(A2,B2)** rat. **(C,D)** CA1 region of the dorsal **(C)** and ventral **(D)** hippocampus, including the strata oriens (Or), pyramidale (Py) radiatum (Rad) and lacunosum-moleculare (LMol). **(E–G)** superficial (I–III, Sup) and deep (IV–VI) layers of the mPFC, including the prelimbic (PrL, **E**), infralimbic (IL, **F**) and cingulate 1 (Cg1, **G**) cortices. **(H)** Basolateral amygdala (BLA). Values represent mean ± S.E.M. Asterisks indicate statistically significant differences between groups (Control × Stress) after unpaired Student’s *t*-test (^∗^*p* < 0.05; ^∗∗^*p* < 0.01; ^#^0.1 < *p* < 0.05). Scale bars: 15 μm.

We have analyzed PSA-NCAM expression in the neuropil of different telencephalic regions in which this molecule has a considerable expression and in which we have previously detected changes in its expression or in those of molecules involved in inhibitory neurotransmission after chronic stress: the mPFC, the BLA and the dorsal and ventral hippocampus, see Nacher et al. (2013) for review. Chronic stress induced increases in the expression of PSA-NCAM in the CA1 region of the ventral and dorsal hippocampus (Figure 1A–D): In the dorsal hippocampus we observed a significant increase in the stratum lacunosum-moleculare, the layer with higher levels of expression of this molecule (Figure 1C; *p* = 0.006). In the ventral hippocampus we found significant increases in the strata pyramidale and radiatum (Figure 1D; *p* = 0.021, *p* = 0.038). Ten days of restraint stress did not induce significant changes in neuropil PSA-NCAM immunostaining in any of the regions of the mPFC (Figure 1E–G) or in the BLA (Figure 1H).

### Effects of Restraint Stress in the Number of PNN and PV+ Cells

**FIGURE 2:**
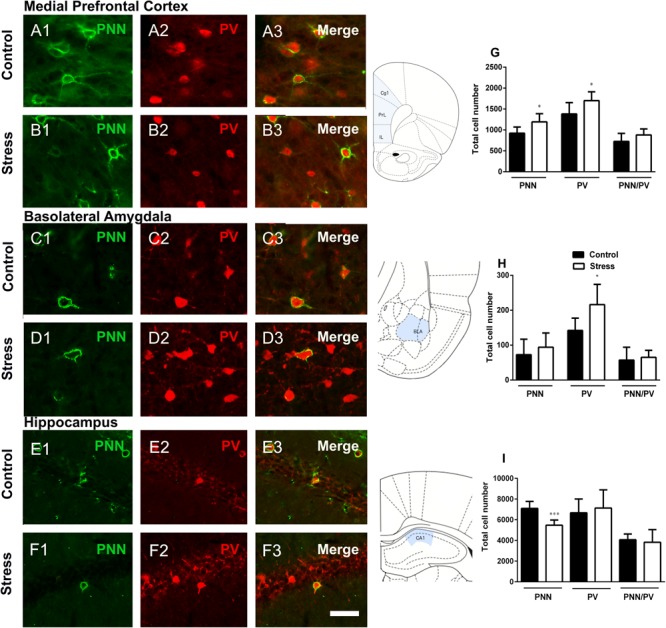
Perineuronal nets (PNN), parvalbumin (PV) expressing neurons and their colocalization in the mPFC, basolateral amygdala and the hippocampal CA1 in control rats and after 10 days of chronic restraint stress. Representative confocal images showing the distribution of PNN (**A1–F1**; green), PV+ somata (**A2–F2**; red) and their colocalization **(A3–F3)** in the mPFC **(A,B)** the basolateral amygdala **(C,D)** and the CA1 region of the hippocampus. Schemes on the right of figures **(A–F)** indicate the studied regions in light blue (modified from Paxinos and Watson, 1998). Graphs on the right side of the figure indicate changes in the total number of PNN, PV+ somata and PV+ somata surrounded by PNN in the mPFC **(G)**, the basolateral amygdala **(H)** and the hippocampus **(I)**. Values represent mean ± S.E.M. Asterisks indicate statistically significant differences between groups (Control × Stress) after unpaired Student’s *t*-test (^∗^*p* < 0.05, ^∗∗∗^*p* < 0.001). PrL: prelimbic cortex, IL: infralimbic cortex, Cg1: cingulate 1 cortex, BLA: basolateral amygdala. Scale bar 15 μm.

The number of PNN (*p* = 0.022) and parvalbumin expressing (PV+) cells (*p* = 0.045) in the mPFC (including the infralimbic, prelimbic and cingulate 1 cortices) were significantly increased in stressed rats when compared to controls. However, there was no significant difference in the number of PV+ cells surrounded by PNN (*p* = 0.146) between the groups (Figure 2A,B,G). The density of PV+ neurons and PNN showed a strong and significant positive linear correlation in the mPFC of control animals (*r* = 0.9118, *p* < 0.01), but this correlation was not significant in stressed individuals (*r* = 0.717, *p* < 0.108). We also investigated whether repeated restraint stress altered the number of PV+ cells and PNN in the BLA. The number of PV+ cells in this amygdaloid region was significantly increased in stressed rats (*p* = 0.023). There were no significant differences in the number of PNN (*p* = 0.407) or PNN/ PV+ cells (*p* = 0.653) (Figure 2C,D,H). The number of PV+ and PNN neurons showed a significant positive linear correlation in the BLA of control animals (*r* = 0.804, *p* < 0.05), but such correlation did not exist in stressed individuals (*r* = –0.04, *p* < 0.941).

The analysis of the number of PNN in the hippocampal CA1 region revealed a significant decrease in stressed rats when compared to controls (*p* = 0.0008). There were no significant differences in the number of PV+ cells (*p* = 0.626) or PNN/PV+ cells (*p* = 0.670) (Figure 2E,F,I). The number of PV+ neurons and PNN did not show a significant linear correlation neither in the CA1 of control animals (*r* = –0.407, *p* < 0.422), nor in that of stressed individuals (*r* = 0.681, *p* < 0.136).

**FIGURE 3:**
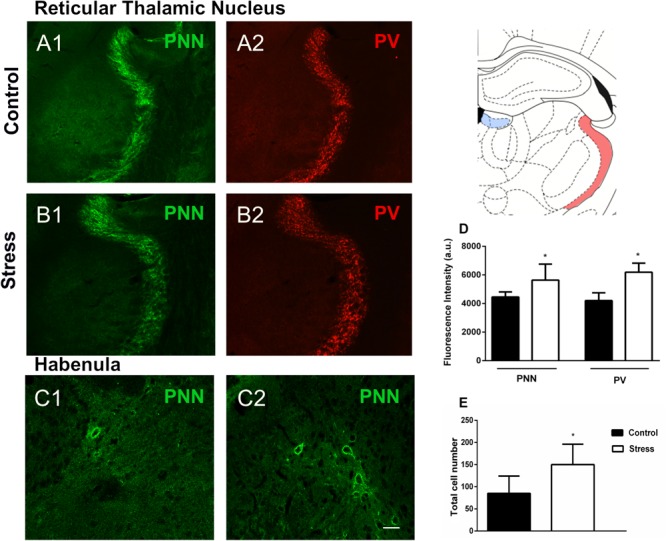
Perineuronal nets (PNN), parvalbumin (PV) expressing neurons and their colocalization in the reticular thalamic nucleus and the habenula in control rats and after 10 days of chronic restraint stress. **(A,B)** Representative confocal images showing the distribution of PNN (**A1,B1**; green) and PV+ somata (**A2,B2**; red) in the reticular thalamic nucleus of control **(A)** and stressed **(B)** rats. **(C)** Confocal images showing the distribution of PNN (green) in the habenula of control **(C1)** and stressed **(C2)** rats; no PV immunoreactive somata could be detected in the habenula, neither in control nor in stressed animals. **(D,E)**: Graphs on the right side of the figure indicate changes in the fluorescence intensity of PNN and PV stainings in the reticular thalamic nucleus **(D)** and of the total numbers of PNN in the habenula **(E)**. Schemes on the top right of the figure indicate the studied regions in light red (reticular thalamic nucleus) and light blue (habenula) (modified from Paxinos and Watson, 1998). Values represent mean ± S.E.M. Asterisks indicate statistically significant differences between groups (Control × Stress) after unpaired Student’s *t*-test (^∗^*p* < 0.05). Scale bar 15 μm.

We also studied whether repeated restraint stress affected PNN and PV+ cells in the TRN. By analyzing the intensity of fluorescence, we found that stress clearly increased the immunoreactivity of PNN (*p* = 0.033) and PV (*p* = 0.0002) (Figure 3A,B,D). Using the same methodology, we analyzed WFA fluorescence in the hippocampal CA2 and did not find significant differences (*p* = 0.448) after chronic stress. In accordance with previous reports (Smith et al., 1987; Li et al., 2011; Meye et al., 2013) we failed to detect parvalbumin expressing interneurons in the habenula, although some PNN could be observed and quantified. The number of these PV–/PNN was significantly increased (*p* = 0.025) after 10 days of chronic restraint stress (Figure 3C,E).

### Effects of Restraint Stress on PV Immunoreactive Puncta on the Perisomatic Region of mPFC and Basolateral Amygdala Excitatory Neurons

**FIGURE 4:**
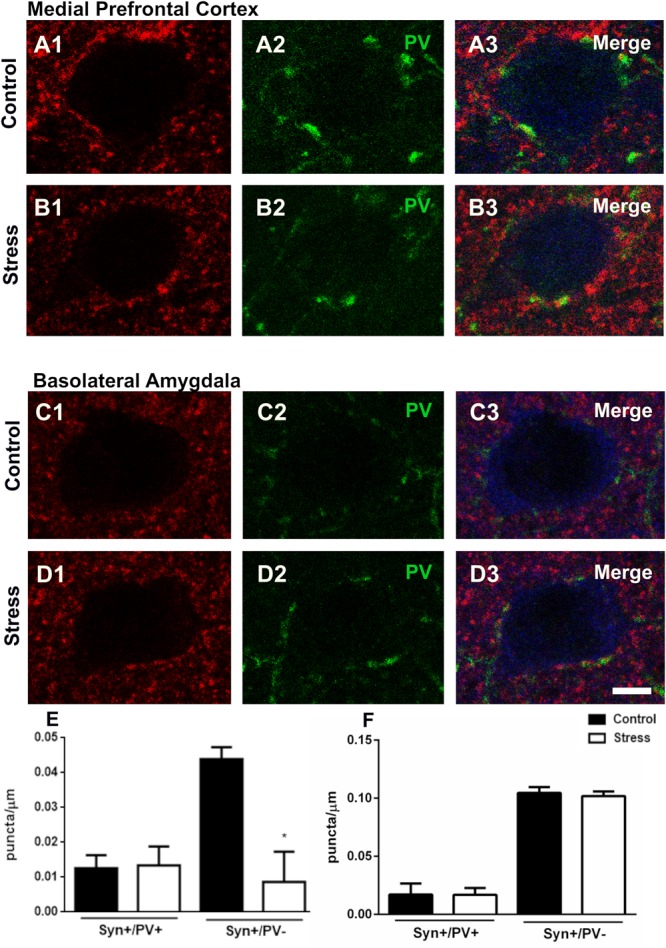
Confocal microscopic analysis of parvalbumin (PV) and synaptophysin (SYN) expressing puncta in the perisomatic region of excitatory neurons in the mPFC and basolateral amygdala in control rats and after 10 days of chronic restraint stress. Single confocal planes of principal neuron somata (immunolabeled for CaMKII-α; blue) showing perisomatic puncta immunoreactive for SYN (**A1–D1**; red) and PV (**A2–D2**; green) and their colocalization **(A3–D3)** in the mPFC **(A,B)** and the basolateral amygdala **(C,D)**. Graphs on the bottom of the figure show changes in the linear density (number of puncta/μm of soma perimeter) of synaptophysin expressing perisomatic puncta with (Syn+/PV+) or without (Syn+/PV–) PV coexpression (**E**: mPFC; **F**: BLA). Values represent mean ± S.E.M. Asterisks indicate statistically significant differences between groups (Control × Stress) after unpaired Student’s *t*-test (^∗^*p* < 0.05). Scale bar 5 μm.

Since repeated restraint stress altered the number of PV+ interneurons in the mPFC and the BLA and given the importance of these cells on the perisomatic inhibition of excitatory neurons, we decided to analyze the density of synaptophysin (SYN) expressing puncta with or without PV immunoreactivity surrounding the somata of these principal neurons. The excitatory neuronal somata were identified by the presence of CAMKII immunoreactivity. In the mPFC repeated restraint stress did not change the density of SYN+/ PV+ puncta but decreased significantly that of PV–/SYN expressing puncta (*p* = 0.019), (Figure 4A,B,E). Chronic stress did not significantly change the density of perisomatic puncta on excitatory neurons of the BLA (Figure 4C,D,F).

## Discussion

The main objective of this study was to evaluate the effects of chronic stress on molecules related to interneuronal plasticity in different regions of the adult CNS. Our results reveal only local effects on PSA-NCAM expression in the hippocampal CA1 region. By contrast, they show wider and more profound alterations in the number of PNN, which in some cases are associated to PV+ and in some others appear to be independent from these interneurons. Interestingly, these alterations in PNN are not only found in the commonly studied regions in chronic stress models, but also in less explored areas, such as the habenula or the reticular thalamic nucleus.

We first evaluated the expression of PSA-NCAM, since this molecule is expressed by a subpopulation of interneurons in the adult telencephalon and regulates their structure and connectivity (Gomez-Climent et al., 2011). We have only found a considerable increase in PSA-NCAM expression in the stratum lacunosum-moleculare of CA1. It is probable that these changes affect mainly O-LM cells, which are interneurons that project specifically to this layer and which morphology and connectivity is particularly regulated by PSA (Guirado et al., 2014). Moreover, changes in the structure of these interneurons and of GAD67 expression in CA1 have been observed in mice subjected to chronic stress (Gilabert-Juan et al., 2017). The presence of higher levels of PSA-NCAM expression in these cells may thus increase their insulation, leading to decreases in dendritic complexity and reduced connectivity (Gomez-Climent et al., 2011). However, in the CA1 PSA-NCAM is not exclusively associated to inhibitory elements (Gomez-Climent et al., 2011) and, consequently, changes in the expression of this molecule might be also affecting the connectivity of principal neurons.

The other focus of our study was on parvalbumin expressing interneurons and their relationship with PNN. Our results showing an increase in the number of PV expressing neurons in the mPFC or the BLA are apparently in contrast with previous reports describing decreases in prefrontocortical PV+ somata after chronic stress [5 weeks of chronic unpredictable stress (Banasr et al., 2017)], although some were only found in anhedonic animals (9 weeks of chronic mild stress) (Czeh et al., 2018). Another report, using 2 weeks of chronic unpredictable stress or 8 weeks of chronic mild stress, did not find changes in the density of PV+ somata in the mPFC of Wistar rats (Zadrozna et al., 2011). However, in agreement with our results, recent studies with shorter periods (2 weeks) of chronic stress have found significant increases in the number of PV+ cells and PV mRNA in the mPFC of adult female mice and in the density of excitatory puncta on the perisomatic region of PV cells in both sexes (Shepard et al., 2016; Shepard and Coutellier, 2018). Furthermore, Filipovic et al. (2018) and Todorovic et al. (2019) demonstrated that chronic social isolation leads to a decreased number of PV+ cells in the mPFC and hippocampus of adult male Wistar rats.

Additionally, chronic variable stress for 2 weeks in the same rat strain used in our experiment increased the inhibition of mPFC pyramidal cells and the density of inhibitory puncta around the somata of these principal neurons (McKlveen et al., 2016). In contrast with these results, we have failed to find decreases in the density of puncta coexpressing PV and synaptophysin (SYN) in our stressed animals. However, the density of PV–SYN+ puncta decreased markedly, which may indicate a reduction in other type of perisomatic synapses, more likely of those coming from cholecystokinin (CCK) expressing basket interneurons. This is in accordance with the recently found reductions in the number of CCK+ neurons in the mPFC of rats subjected to 9 weeks of chronic mild stress (Czeh et al., 2018).

The variability in the results obtained in our study and the previous ones on the effects of chronic stress on PV+ cells may obey to differences in the rat strain used, the lack of discrimination between different prefrontocortical regions and, particularly, to differences in the duration and nature of the stress protocol employed. It is possible that in animals submitted to shorter stress protocols PV+ interneurons were activated, leading to an increased inhibition on pyramidal neurons. On the other hand, in longer protocols, in which depressive-like behaviors become persistent, these interneurons may show a decrease in their activity and parvalbumin expression. It is also important to note that there is a decrease in excitatory neurotransmission on PV+ interneurons of the mPFC in learned helplessness and that the suppression of the activity of these inhibitory cells promotes the apparition of this depressive-like behavior (Perova et al., 2015). Consequently, the activation of PV+ interneurons in the mPFC in earlier phases of chronic stress may be interpreted as a response to promote the establishment of resilient behaviors, as Shepard and Coutellier (2018), Shepard et al. (2016) and our own data suggest. In fact, previous reports strongly suggest that the end of the 10 days of restraint coincides with a period in which the behavioral effects of stress are starting to be observed (McLaughlin et al., 2007; Reznikov et al., 2008; Grillo et al., 2015).

Similar to what we have observed in the mPFC, we also found an increase in the number of PV+ cells in the BLA of our stressed rats. Reznikov et al. (2008), using the same stress paradigm employed in the present study did not find changes in the density of PV+ cells in the BLA. This discrepancy may arise from differences in the methodology employed for quantification: We estimated the total number of PV+ interneurons in the whole BLA using a modified version of the fractionator method (West, 1993; Nacher et al., 2002), while cell densities were calculated specifically from the medial-caudal extent of the anterior subdivision of this amygdaloid region (Reznikov et al., 2008). It has to be noted, however, that Reznikov et al. (2008) observed a marked increase in the percentage of PV+ cells displaying c-Fos immunoreactivity in their nuclei, which also suggest an activation of these interneurons in this stress protocol.

We do not believe that the increase in PV+ interneurons is due to the incorporations of new cells to the circuitry. We think that the increase in number reflects an increase in the expression of this calcium binding protein and that some interneurons that expressed very low levels of PV (and thus were not detected in control animals), increase their PV expression and become detectable in the stressed animals. A similar switch toward high parvalbumin expression has been observed in the hippocampus after fear conditioning (Donato et al., 2013). Previous results from our laboratory have also described this phenomenon in the infralimbic cortex after social isolation rearing (Castillo-Gomez et al., 2017) or after treatment with a dopamine D2 receptor agonist (Castillo-Gomez et al., 2011). The decrease in the number of PV expressing neurons is not due to a decrease in the volume of the neuropil because for applying the modified version of the fractionator method we calculated the volumes of the areas and found no differences between groups.

There is another report studying the effects of chronic stress on PNN during adulthood, using a social defeat-induced persistent stress (SDPS) in Wistar rats, a very long-lasting protocol, which induces persistent depressive-like behavior (Riga et al., 2017). In this study the authors found a marked increase in the density of PNN in the hippocampal CA1 and specifically of those surrounding PV+ interneurons. These results are in sharp contrast with our present findings in this hippocampal region, where we observe a significant decreased density of PNN. However, we did not find changes in the number of PV+ neurons surrounded by PNN. In fact, we do not observe significant linear correlations between PV+ cells and PNN in the CA1, neither in controls nor in stressed animals, suggesting that the reduction in PNN that we observe may be due to decreases in PNN associated to other cell types, more likely excitatory neurons. Although Riga et al. (2017) found that in Wistar rats more than 90% of PNN in the CA1 were associated to PV+ neurons, and other authors found around 80% in Long-Evans rats (Lensjo et al., 2017), in our study we only found around 60%. Interestingly, although we find positive correlations between the numbers of PV+ interneurons and PNN in the mPFC and the BLA in control animals, such correlations did not exist in stressed individuals, which suggest that the changes that we observe after stress in the PV subpopulation of interneurons may be independent of the presence of PNN surrounding them.

In the case of the TRN the effects of stress should mainly affect PV+ cells, since these neurons are a large proportion of the cellular constituents of this thalamic nucleus and most, if not all of them, are surrounded by PNN. Our results on this nucleus are particularly interesting because of their robustness and because they clearly show for the first time the involvement of this thalamic region in the response to chronic stress during adulthood. A previous study already highlighted the putative involvement of this nucleus in the response to acute stress, due to an important increase in c-Fos expression (Ons et al., 2004). Further research in this region needs to be done to understand how the structure, neurotransmission and connectivity of its neurons is affected by stress.

To our knowledge there is only one study describing PNN in the habenula, focused on their postnatal development in mice (Horii-Hayashi et al., 2015); the present data is the first to describe their presence in adult animals and their modulation by chronic stress. We do not know yet the phenotype of the habenular neurons surrounded by these PNN, but most likely they are excitatory neurons, since interneurons are very rare in this thalamic region (Smith et al., 1987; Li et al., 2011; Meye et al., 2013). The present results support previous work pointing out a prominent role of the habenula in the response to chronic stress (Wirtshafter et al., 1994; Aizawa et al., 2013; Jacinto et al., 2017).

The significance of the increases in PNN numbers that we have found in the present study is still far from clear. It has been proposed that PNN may act by protecting fast spiking neurons from oxidative stress (Cabungcal et al., 2013). Since chronic stress induces high levels of reactive oxygen species (Madrigal et al., 2006), the increases in PNN at this early phase of stress may represent an attempt of the neurons to counteract the effects of this oxidative stress. Studies using longer stress duration are needed to know whether at later stages of chronic stress the numbers of PNN and PV+ cells decrease or return to control levels. Transgenic mice with impaired redox regulation show decreased number of PV+ neurons and PNN in their PFC and TRN (Cabungcal et al., 2013; Steullet et al., 2017). Interestingly, patients suffering from schizophrenia, in which stress is known to be a precipitating factor, also show reductions in the density of PV+ cells and PNN in this two regions (Mauney et al., 2013; Steullet et al., 2017). PNN may also act by influencing synaptic plasticity, constituting a physical barrier to stabilize synaptic contacts or to prevent the formation of new contacts through the expression of inhibitory molecules (Nowicka et al., 2009; Sorg et al., 2016).

In conclusion, our data demonstrate that a short chronic stress can induce changes in molecules involved in interneuronal plasticity, not only in “classical” regions involved in the stress response, but also in the habenula or the TRN, a region in which we have described for the first time alterations induced by this aversive experience. This supports the hypothesis that alterations in inhibitory networks and of molecules involved in their plasticity, particularly the PNN, underlie the impact of stress in the CNS and may participate in the development of certain psychiatric disorders.

## Author Contributions

JN, AP, and MG-C designed the experiments. AP, MG-C, CB-F, YC, and HC performed the experiments. AP, RG, and JN wrote the manuscript. JN supervised the experiments and wrote the final version of the manuscript.

## Conflict of Interest Statement

The authors declare that the research was conducted in the absence of any commercial or financial relationships that could be construed as a potential conflict of interest.

## References

[B1] Aizawa H. Cui W. Tanaka K. Okamoto H. (2013). Hyperactivation of the habenula as a link between depression and sleep disturbance. *Front. Hum. Neurosci.* 7:826. 10.3389/fnhum.2013.00826 24339810 PMC3857532

[B2] Banasr M. Lepack A. Fee C. Duric V. Maldonado-Aviles J. DiLeone R. (2017). Characterization of GABAergic marker expression in the chronic unpredictable stress model of depression. *Chronic Stress* 1. 10.1177/2470547017720459 28835932 PMC5565173

[B3] Banerjee S. B. Gutzeit V. A. Baman J. Aoued H. S. Doshi N. K. Liu R. C. (2017). Perineuronal nets in the adult sensory cortex are necessary for fear learning. *Neuron* 95 169–179.e3. 10.1016/j.neuron.2017.06.007 28648500 PMC5548423

[B4] Belleau E. L. Treadway M. T. Pizzagalli D. A. (2018). The impact of stress and major depressive disorder on hippocampal and medial prefrontal cortex morphology. *Biol. Psychiatry* 85 443–453. 10.1016/j.biopsych.2018.09.031 30470559 PMC6380948

[B5] Berretta S. Pantazopoulos H. Markota M. Brown C. Batzianouli E. T. (2015). Losing the sugar coating: potential impact of perineuronal net abnormalities on interneurons in schizophrenia. *Schizophr. Res.* 167 18–27. 10.1016/j.schres.2014.12.040 25601362 PMC4504843

[B6] Cabungcal J. H. Steullet P. Morishita H. Kraftsik R. Cuenod M. Hensch T. K. (2013). Perineuronal nets protect fast-spiking interneurons against oxidative stress. *Proc. Natl. Acad. Sci. U.S.A.* 110 9130–9135. 10.1073/pnas.1300454110 23671099 PMC3670388

[B7] Carulli D. Pizzorusso T. Kwok J. C. Putignano E. Poli A. Forostyak S. (2010). Animals lacking link protein have attenuated perineuronal nets and persistent plasticity. *Brain* 133(Pt 8) 2331–2347. 10.1093/brain/awq145 20566484

[B8] Castillo-Gomez E. Perez-Rando M. Belles M. Gilabert-Juan J. Llorens J. V. Carceller H. (2017). Early social isolation stress and perinatal NMDA receptor antagonist treatment induce changes in the structure and neurochemistry of inhibitory neurons of the adult amygdala and prefrontal cortex. *eNeuro* 4:ENEURO.0034-17.2017. 10.1523/ENEURO.0034-17.2017 28466069 PMC5411163

[B9] Castillo-Gomez E. Varea E. Blasco-Ibanez J. M. Crespo C. Nacher J. (2011). Polysialic acid is required for dopamine D2 receptor-mediated plasticity involving inhibitory circuits of the rat medial prefrontal cortex. *PLoS One* 6:e29516. 10.1371/journal.pone.0029516 22216301 PMC3247286

[B10] Castillo-Gomez E. Varea E. Blasco-Ibanez J. M. Crespo C. Nacher J. (2016). Effects of chronic dopamine D2R agonist treatment and polysialic acid depletion on dendritic spine density and excitatory neurotransmission in the mPFC of adult rats. *Neural Plast.* 2016:1615363. 10.1155/2016/1615363 27110404 PMC4821975

[B11] Czeh B. Simon M. van der Hart M. G. Schmelting B. Hesselink M. B. Fuchs E. (2005). Chronic stress decreases the number of parvalbumin-immunoreactive interneurons in the hippocampus: prevention by treatment with a substance P receptor (NK1) antagonist. *Neuropsychopharmacology* 30 67–79. 10.1038/sj.npp.1300581 15470372

[B12] Czeh B. Vardya I. Varga Z. Febbraro F. Csabai D. Martis L. S. (2018). Long-term stress disrupts the structural and functional integrity of GABAergic neuronal networks in the medial prefrontal cortex of rats. *Front. Cell. Neurosci.* 12:148. 10.3389/fncel.2018.00148 29973870 PMC6020798

[B13] Czeh B. Varga Z. K. Henningsen K. Kovacs G. L. Miseta A. Wiborg O. (2015). Chronic stress reduces the number of GABAergic interneurons in the adult rat hippocampus, dorsal-ventral and region-specific differences. *Hippocampus* 25 393–405. 10.1002/hipo.22382 25331166

[B14] Di Cristo G. Chattopadhyaya B. Kuhlman S. J. Fu Y. Belanger M. C. Wu C. Z. (2007). Activity-dependent PSA expression regulates inhibitory maturation and onset of critical period plasticity. *Nat. Neurosci.* 10 1569–1577. 10.1038/nn2008 18026099

[B15] Donato F. Rompani S. B. Caroni P. (2013). Parvalbumin-expressing basket-cell network plasticity induced by experience regulates adult learning. *Nature* 504 272–276. 10.1038/nature12866 24336286

[B16] Drevets W. C. Price J. L. Furey M. L. (2008). Brain structural and functional abnormalities in mood disorders: implications for neurocircuitry models of depression. *Brain Struct. Funct.* 213 93–118. 10.1007/s00429-008-0189-x 18704495 PMC2522333

[B17] Filipovic D. Stanisavljevic A. Jasnic N. Bernardi R. E. Inta D. Peric I. (2018). Chronic treatment with fluoxetine or clozapine of socially isolated rats prevents subsector-specific reduction of parvalbumin immunoreactive cells in the hippocampus. *Neuroscience* 371 384–394. 10.1016/j.neuroscience.2017.12.020 29275206

[B18] Gilabert-Juan J. Bueno-Fernandez C. Castillo-Gomez E. Nacher J. (2017). Reduced interneuronal dendritic arborization in CA1 but not in CA3 region of mice subjected to chronic mild stress. *Brain Behav.* 7:e00534. 10.1002/brb3.534 28239515 PMC5318357

[B19] Gilabert-Juan J. Castillo-Gomez E. Guirado R. Molto M. D. Nacher J. (2013). Chronic stress alters inhibitory networks in the medial prefrontal cortex of adult mice. *Brain Struct. Funct.* 218 1591–1605. 10.1007/s00429-012-0479-1 23179864

[B20] Gilabert-Juan J. Castillo-Gomez E. Perez-Rando M. Molto M. D. Nacher J. (2011). Chronic stress induces changes in the structure of interneurons and in the expression of molecules related to neuronal structural plasticity and inhibitory neurotransmission in the amygdala of adult mice. *Exp. Neurol.* 232 33–40. 10.1016/j.expneurol.2011.07.009 21819983

[B21] Gogolla N. Caroni P. Lüthi A. Herry C. (2009). Perineuronal nets protect fear memories from erasure. *Science* 325 1258–1261. 10.1126/science.1174146 19729657

[B22] Gomez-Climent M. A. Guirado R. Castillo-Gomez E. Varea E. Gutierrez-Mecinas M. Gilabert-Juan J. (2011). The polysialylated form of the neural cell adhesion molecule (PSA-NCAM) is expressed in a subpopulation of mature cortical interneurons characterized by reduced structural features and connectivity. *Cereb Cortex* 21 1028–1041. 10.1093/cercor/bhq177 20843898

[B23] Grillo C. A. Risher M. Macht V. A. Bumgardner A. L. Hang A. Gabriel C. (2015). Repeated restraint stress-induced atrophy of glutamatergic pyramidal neurons and decreases in glutamatergic efflux in the rat amygdala are prevented by the antidepressant agomelatine. *Neuroscience* 284 430–443. 10.1016/j.neuroscience.2014.09.047 25280788

[B24] Guirado R. Perez-Rando M. Sanchez-Matarredona D. Castillo-Gomez E. Liberia T. Rovira-Esteban L. (2014). The dendritic spines of interneurons are dynamic structures influenced by PSA-NCAM expression. *Cereb Cortex* 24 3014–3024. 10.1093/cercor/bht156 23780867

[B25] Gundersen H. J. Jensen E. B. (1987). The efficiency of systematic sampling in stereology and its prediction. *J. Microsc.* 147(Pt 3) 229–263. 10.1111/j.1365-2818.1987.tb02837.x 3430576

[B26] Hartig W. Brauer K. Bruckner G. (1992). Wisteria floribunda agglutinin-labelled nets surround parvalbumin-containing neurons. *Neuroreport* 3 869–872. 10.1097/00001756-199210000-00012 1421090

[B27] Horii-Hayashi N. Sasagawa T. Matsunaga W. Nishi M. (2015). Development and structural variety of the chondroitin sulfate proteoglycans-contained extracellular matrix in the mouse brain. *Neural Plast.* 2015:256389. 10.1155/2015/256389 26649203 PMC4663360

[B28] Jacinto L. R. Mata R. Novais A. Marques F. Sousa N. (2017). The habenula as a critical node in chronic stress-related anxiety. *Exp. Neurol.* 289 46–54. 10.1016/j.expneurol.2016.12.003 27940019

[B29] Lensjo K. K. Christensen A. C. Tennoe S. Fyhn M. Hafting T. (2017). Differential expression and cell-type specificity of perineuronal nets in hippocampus, medial entorhinal cortex, and visual cortex examined in the rat and mouse. *eNeuro* 4:ENEURO.0379-16.2017. 10.1523/ENEURO.0379-16.2017 28593193 PMC5461557

[B30] Li B. Piriz J. Mirrione M. Chung C. Proulx C. D. Schulz D. (2011). Synaptic potentiation onto habenula neurons in the learned helplessness model of depression. *Nature* 470 535–539. 10.1038/nature09742 21350486 PMC3285101

[B31] Madrigal J. L. Garcia-Bueno B. Caso J. R. Perez-Nievas B. G. Leza J. C. (2006). Stress-induced oxidative changes in brain. *CNS Neurol. Disord. Drug Targets* 5 561–568. 10.2174/187152706778559327 17073658

[B32] Mauney S. A. Athanas K. M. Pantazopoulos H. Shaskan N. Passeri E. Berretta S. (2013). Developmental pattern of perineuronal nets in the human prefrontal cortex and their deficit in schizophrenia. *Biol. Psychiatry* 74 427–435. 10.1016/j.biopsych.2013.05.007 23790226 PMC3752333

[B33] McEwen B. S. (2000). The neurobiology of stress: from serendipity to clinical relevance. *Brain Res.* 886 172–189. 10.1016/s0006-8993(00)02950-4 11119695

[B34] McKlveen J. M. Morano R. L. Fitzgerald M. Zoubovsky S. Cassella S. N. Scheimann J. R. (2016). Chronic stress increases prefrontal inhibition: a mechanism for stress-induced prefrontal dysfunction. *Biol. Psychiatry* 80 754–764. 10.1016/j.biopsych.2016.03.2101 27241140 PMC5629635

[B35] McLaughlin K. J. Gomez J. L. Baran S. E. Conrad C. D. (2007). The effects of chronic stress on hippocampal morphology and function: an evaluation of chronic restraint paradigms. *Brain Res.* 1161 56–64. 10.1016/j.brainres.2007.05.042 17603026 PMC2667378

[B36] Meye F. J. Lecca S. Valentinova K. Mameli M. (2013). Synaptic and cellular profile of neurons in the lateral habenula. *Front. Hum. Neurosci.* 7:860. 10.3389/fnhum.2013.00860 24379770 PMC3863943

[B37] Nacher J. Alonso-Llosa G. Rosell D. McEwen B. (2002). PSA-NCAM expression in the piriform cortex of the adult rat. Modulation by NMDA receptor antagonist administration. *Brain Res.* 927 111–121. 10.1016/s0006-8993(01)03241-3 11821005

[B38] Nacher J. Guirado R. Castillo-Gomez E. (2013). Structural plasticity of interneurons in the adult brain: role of PSA-NCAM and implications for psychiatric disorders. *Neurochem. Res.* 38 1122–1133. 10.1007/s11064-013-0977-4 23354722

[B39] Nowicka D. Soulsby S. Skangiel-Kramska J. Glazewski S. (2009). Parvalbumin-containing neurons, perineuronal nets and experience-dependent plasticity in murine barrel cortex. *Eur. J. Neurosci.* 30 2053–2063. 10.1111/j.1460-9568.2009.06996.x 20128844

[B40] Ons S. Marti O. Armario A. (2004). Stress-induced activation of the immediate early gene Arc (activity-regulated cytoskeleton-associated protein) is restricted to telencephalic areas in the rat brain: relationship to c-fos mRNA. *J. Neurochem.* 89 1111–1118. 10.1111/j.1471-4159.2004.02396.x 15147503

[B41] Pantazopoulos H. Berretta S. (2016). In sickness and in health: perineuronal nets and synaptic plasticity in psychiatric disorders. *Neural Plast.* 2016:9847696. 10.1155/2016/9847696 26839720 PMC4709762

[B42] Paxinos G. Watson C. (1998). *The Rat Brain in Stereotaxic Coordinates.* Sydney: Academic Press. 10.4236/pp.2011.24049

[B43] Perova Z. Delevich K. Li B. (2015). Depression of excitatory synapses onto parvalbumin interneurons in the medial prefrontal cortex in susceptibility to stress. *J. Neurosci.* 35 3201–3206. 10.1523/JNEUROSCI.2670-14.2015 25698754 PMC4331634

[B44] Pesarico A. P. Rosa S. G. Martini F. Goulart T. A. Zeni G. Nogueira C. W. (2017). Brain-derived neurotrophic factor signaling plays a role in resilience to stress promoted by isoquinoline in defeated mice. *J. Psychiatr. Res.* 94 78–87. 10.1016/j.jpsychires.2017.06.012 28688339

[B45] Radley J. Morilak D. Viau V. Campeau S. (2015). Chronic stress and brain plasticity: mechanisms underlying adaptive and maladaptive changes and implications for stress-related CNS disorders. *Neurosci. Biobehav. Rev.* 58 79–91. 10.1016/j.neubiorev.2015.06.018 26116544 PMC4684432

[B46] Reznikov L. R. Reagan L. P. Fadel J. R. (2008). Activation of phenotypically distinct neuronal subpopulations in the anterior subdivision of the rat basolateral amygdala following acute and repeated stress. *J. Comp. Neurol.* 508 458–472. 10.1002/cne.21687 18335544

[B47] Riga D. Kramvis I. Koskinen M. K. van Bokhoven P. van der Harst J. E. Heistek T. S. (2017). Hippocampal extracellular matrix alterations contribute to cognitive impairment associated with a chronic depressive-like state in rats. *Sci. Transl. Med.* 9:eaai8753. 10.1126/scitranslmed.aai8753 29263233

[B48] Roozendaal B. McEwen B. S. Chattarji S. (2009). Stress, memory and the amygdala. *Nat. Rev. Neurosci.* 10 423–433. 10.1038/nrn2651 19469026

[B49] Sachs B. D. Tran H. L. Folse E. Caron M. G. (2018). Brain-region-specific molecular responses to maternal separation and social defeat stress in mice. *Neuroscience* 373 122–136. 10.1016/j.neuroscience.2018.01.018 29341883 PMC5816704

[B50] Sandi C. (2004). Stress, cognitive impairment and cell adhesion molecules. *Nat. Rev. Neurosci.* 5 917–930. 10.1038/nrn1555 15550947

[B51] Schindelin J. Arganda-Carreras I. Frise E. Kaynig V. Longair M. Pietzsch T. (2012). Fiji: an open-source platform for biological-image analysis. *Nat. Methods* 9 676–682. 10.1038/nmeth.2019 22743772 PMC3855844

[B52] Shepard R. Coutellier L. (2018). Changes in the prefrontal glutamatergic and parvalbumin systems of mice exposed to unpredictable chronic stress. *Mol. Neurobiol.* 55 2591–2602. 10.1007/s12035-017-0528-0 28421533

[B53] Shepard R. Page C. E. Coutellier L. (2016). Sensitivity of the prefrontal GABAergic system to chronic stress in male and female mice: relevance for sex differences in stress-related disorders. *Neuroscience* 332 1–12. 10.1016/j.neuroscience.2016.06.038 27365172

[B54] Smith Y. Seguela P. Parent A. (1987). Distribution of GABA-immunoreactive neurons in the thalamus of the squirrel monkey (*Saimiri sciureus*). *Neuroscience* 22 579–591. 10.1016/0306-4522(87)90355-1 3670598

[B55] Soleman S. Filippov M. A. Dityatev A. Fawcett J. W. (2013). Targeting the neural extracellular matrix in neurological disorders. *Neuroscience* 253 194–213. 10.1016/j.neuroscience.2013.08.050 24012743

[B56] Sorg B. A. Berretta S. Blacktop J. M. Fawcett J. W. Kitagawa H. Kwok J. C. (2016). Casting a wide net: role of perineuronal nets in neural plasticity. *J. Neurosci.* 36 11459–11468. 10.1523/JNEUROSCI.2351-16.2016 27911749 PMC5125213

[B57] Steullet P. Cabungcal J. H. Bukhari S. A. Ardelt M. I. Pantazopoulos H. Hamati F. (2017). The thalamic reticular nucleus in schizophrenia and bipolar disorder: role of parvalbumin-expressing neuron networks and oxidative stress. *Mol. Psychiatry* 23 2057–2065. 10.1038/mp.2017.230 29180672 PMC5972042

[B58] Todorovic N. Micic B. Schwirtlich M. Stevanovic M. Filipovic D. (2019). Subregion-specific protective effects of fluoxetine and clozapine on parvalbumin expression in medial prefrontal cortex of chronically isolated rats. *Neuroscience* 396 24–35. 10.1016/j.neuroscience.2018.11.008 30448452

[B59] Ueno H. Suemitsu S. Murakami S. Kitamura N. Wani K. Matsumoto Y. (2018). Juvenile stress induces behavioral change and affects perineuronal net formation in juvenile mice. *BMC Neurosci.* 19:41. 10.1186/s12868-018-0442-z 30012101 PMC6048828

[B60] Varea E. Blasco-Ibanez J. M. Gomez-Climent M. A. Castillo-Gomez E. Crespo C. Martinez-Guijarro F. J. (2007). Chronic fluoxetine treatment increases the expression of PSA-NCAM in the medial prefrontal cortex. *Neuropsychopharmacology* 32 803–812. 10.1038/sj.npp.1301183 16900104

[B61] Varea E. Guirado R. Gilabert-Juan J. Marti U. Castillo-Gomez E. Blasco-Ibanez J. M. (2012). Expression of PSA-NCAM and synaptic proteins in the amygdala of psychiatric disorder patients. *J. Psychiatr. Res.* 46 189–197. 10.1016/j.jpsychires.2011.10.011 22099865

[B62] West M. J. (1993). New stereological methods for counting neurons. *Neurobiol. Aging* 14 275–285. 10.1016/0197-4580(93)90112-o 8367009

[B63] Wirtshafter D. Asin K. E. Pitzer M. R. (1994). Dopamine agonists and stress produce different patterns of Fos-like immunoreactivity in the lateral habenula. *Brain Res.* 633 21–26. 10.1016/0006-8993(94)91517-2 8137158

[B64] Zadrozna M. Nowak B. Lason-Tyburkiewicz M. Wolak M. Sowa-Kucma M. Papp M. (2011). Different pattern of changes in calcium binding proteins immunoreactivity in the medial prefrontal cortex of rats exposed to stress models of depression. *Pharmacol. Rep.* 63 1539–1546. 10.1016/s1734-1140(11)70718-6 22358102

